# Curcumin Suppresses Aldosterone-Induced CRP Generation in Rat Vascular Smooth Muscle Cells via Interfering with the ROS-ERK1/2 Signaling Pathway

**DOI:** 10.1155/2020/3245653

**Published:** 2020-08-07

**Authors:** Xiaolu Zhang, Juntian Liu, Xiaoming Pang, Jingjing Zhao, Shouzhu Xu

**Affiliations:** ^1^Department of Basic Medicine, Hebei University of Chinese Medicine, 3 Xingyuan Road, Shijiazhuang, China; ^2^Hebei Key Laboratory of Chinese Medicine Research on Cardio-cerebrovascular Disease, 3 Xingyuan Road, Shijiazhuang, China; ^3^Department of Pharmacology, Xi'an Jiaotong University School of Medicine, 76 West Yanta Road, Xi'an, China

## Abstract

Aldosterone regulates the initiation and development of atherosclerosis which is identified as a chronic inflammatory disease by promoting the generation of C-reactive protein in vascular smooth muscle cells. Curcumin is the most active ingredient of turmeric with anti-inflammation and antioxidation effects. Here, the effect of curcumin on aldosterone-induced C-reactive protein generation in vascular smooth muscle and the molecular mechanisms involved were explored. Primary rat vascular smooth muscle cells and hyperaldosteronism model rats were used in this study. The amount of C-reactive protein, reactive oxygen species, and the signaling pathway-related molecules generated were estimated. We found that curcumin inhibited aldosterone-induced C-reactive protein generation in vascular smooth muscle cells by interfering with the reactive oxygen species-ERK1/2 signal pathway. The results provide new evidence for the potential anti-inflammatory and cardiovascular protective effects of curcumin.

## 1. Introduction

Ischemic cardiocerebrovascular disease is a common disease that seriously deteriorates human health. Atherosclerosis, its main pathological basis, is considered to be a chronic disease of the blood vessels associated with hyperlipidemia. In recent years, increasing epidemiological and experimental studies have shown that inflammation plays a crucial role in different stages of atherosclerosis progression [1].

Detection of inflammatory factors can be used to diagnose and estimate the severity of inflammatory diseases. The nonspecific inflammation factor C-reactive protein (CRP) is an important inflammatory factor in atherosclerosis. CRP not only predicts cardiovascular events but also serves as an independent risk factor of cardiovascular conditions [2]. In addition, CRP participates in the pathogenesis of atherosclerosis through multiple ways such as induction of vascular endothelial dysfunction and promoting adhesion of monocyte/macrophage to the vascular endothelium, inter alia [3].

Multiple reasons may contribute to the overactivation of the renin-angiotensin-aldosterone system (RAAS), such as sympathetic excitation and renal ischemia. Long-term activation of RAAS has been implicated in the development of conditions such as congestive heart failure, systemic hypertension, and chronic kidney disease [4]. As a part of RAAS, aldosterone, secreted from the adrenal cortex, is one of the most important hormones involved in homeostasis of water and electrolytes. Pathologic elevation of the plasma aldosterone level is identified as a risk factor for many cardiovascular diseases [5]. Aldosterone participates in the progression of cardiovascular diseases by inducing vascular contraction, endothelial dysfunction, and the expression of inflammatory cytokines [6]. Our previous study found that aldosterone stimulated CRP generation in rat vascular smooth muscle cells (VSMCs) through the mineralocorticoid receptor- (MR-) reactive oxygen species (ROS) extracellular signal-regulated kinase (ERK1/2) signal pathway [7].

For centuries, turmeric has been used as a natural pigment in the cosmetic, textile industry, and food industry because of its bright yellow color. In China, turmeric is also a traditional Chinese herb used to remove blood stasis, restore menstrual flow, and relieve pain.

Curcumin is the most active component of spice turmeric (also called curry powder), mainly found in turmeric roots (Curcuma longa L.). It has long been studied for its antioxidant, anti-inflammatory, antimutagenic, antimicrobial, and anticancer properties [8]. However, the mechanisms through which it confers cardiovascular protection and anti-inflammatory effects are not well understood. In the present study, we explored whether curcumin can diminish aldosterone-induced CRP generation in VSMCs. We also examined whether the ROS-ERK1/2 signaling pathway mediates the anti-inflammatory and cardiovascular protective effects of curcumin.

## 2. Materials and Methods

### 2.1. Reagents

Dulbecco's high glucose-modified Eagle's medium (DMEM) and fetal bovine serum (FBS) were provided by HyClone (Logan, UT, USA). Curcumin (purity > 95%) was purchased from Xi'an Tianxingjian Natural Bio-products Co. Ltd. (Xi'an, China). Aldosterone and 3-(4,5-dimethylthiazol-2-yl)-2,5-diphenyltetrazolium bromide (MTT) were purchased from Sigma-Aldrich (St. Louis, MO, USA). Rabbit polyclonal CRP antibody and mouse monoclonal glyceraldehyde-3-phosphate dehydrogenase (GAPDH) antibody were provided by Abcam (Cambridge, UK) and CoWin Biotech (Beijing, China), respectively. Mouse monoclonal *α*-actin, ERK1/2, phospho-ERK1/2 antibodies, and 2′, 7′-dichlorodihydrofluororescein diacetate (H_2_DCF-DA) were purchased from Beyotime (Shanghai, China). The iodine [^12^I] aldosterone radioimmunoassay kit was provided by Chemclin (Beijing, China). The enzyme-linked immunosorbent assay (ELISA) kit for CRP detection was from Westtang (Shanghai, China).

### 2.2. Culture of Primary Rat VSMCs

Primary VSMCs were isolated from the thoracic aorta of male Sprague Dawley (SD) rats as described previously (Florian and Watts, 1998). The cell culture conditions were the same as in our previous experiment [7]. The confluent cells at passages 3–8 were chosen for use. Before the experiments, the cells were incubated in a serum-free medium for an additional 24 h.

### 2.3. Assessment of Cell Viability

Cells were incubated for 24 h with 1, 5, 10, 20, 40, 60, 80, and 100 *μ*mol/L of curcumin (in DMSO) or DMEM for control. To examine the viability of VSMCs, the MTT method was performed with the same steps as in the previous study [9].

### 2.4. Reverse Transcription Polymerase Chain Reaction (RT-PCR)

Total RNA was isolated and reverse transcribed into complementary DNA (cDNA) following the same process as in our previous study [7]. The cDNA was amplified using primer pairs specific for rat CRP (sense primer: 5′-CATCTGTGCCACCTGGGAGTC-3′; antisense primer: 5′-AAGCCACCGCCATACGAGTC-3′). GAPDH was amplified as an internal control for normalization (sense primer: 5′-GCAAGTTCAACGGCACAGTCAAG-3′; antisense primer: 5′-ACATACTCAG CACCAGCATCACC-3′). PCR products were separated by electrophoresis on 2% agarose gel. The expression of mRNA was expressed as relative to GADPH mRNA.

### 2.5. Western Blotting Analysis

VSMCs were lysed with the lysis buffer containing the protease inhibitors cocktail and the phosphatase inhibitors. Protein concentration was measured with the BCA protein assay kit. Samples (35 *μ*g each) were separated by 10% SDS-PAGE and transferred to nitrocellulose membranes which were then blocked with 5% BSA. The membranes were incubated with anti-CRP (1 : 150, Abcam), anti-ERK1/2 (1 : 500, Beyotime), anti-phospho-ERK1/2 (1 : 500, Beyotime), or anti-GAPDH (1 : 500, CoWin) antibodies at 4°C overnight. After being washed, the membranes were incubated with a horseradish peroxidase-conjugated second antibody followed by the enhanced chemiluminescence substrate (Pierce Biotechnology, Rockford, IL, USA). The optical density of the bands was scanned and quantified with the Gel Doc 2000 (Bio-Rad). The results are expressed relative to the corresponding loading control GAPDH.

### 2.6. Measurement of ROS in VSMCs

The intracellular ROS were detected by the H_2_DCF-DA fluorescent labeling method [10]. Curcumin at 1.25, 2.5, and 5 *μ*mol/L were added to the medium 1 h before exposure to 10 nmol/L aldosterone for 2 h. Then, H_2_DCF-DA at 10 *μ*mol/L was loaded to the cells for 1 h. Fluorescence images were obtained with a fluorescence microscope at the excitation wavelength of 488 nm and the emission wavelength of 525 nm (Olympus BX51, Japan). Fluorescence intensity was determined and analyzed from the fluorescence images with the Image-pro plus software.

### 2.7. Preparation of Hyperaldosteronism Model Rats

Hyperaldosteronism model rats were established by subchronic administration of aldosterone [11]. Male SD rats (weight = 180–220 g) were randomly divided into control, a hyperaldosteronism model, and two curcumin-treated groups, each group consisting of 8 rats. Rats in the model and curcumin-treated groups were delivered with aldosterone 0.75 *μ*g/h subcutaneously for a period of 4 weeks by using osmotic minipumps. Rats in the control group received a vehicle (PEG 400) in the same manner. Meanwhile, rats in curcumin-treated groups were given curcumin (dissolved in olive oil) intragastrically for 4 weeks at the doses of 100 and 200 mg/kg/day. Rats in control and model groups were given olive oil instead. At the beginning and end of the experiment, systolic blood pressure (SBP) was measured with a noninvasive tail cuff method. In the end, blood was drawn and centrifuged to detect the concentrations of aldosterone and CRP in serum by radioimmunoassay and ELISA, respectively. The thoracic aorta of rats was immediately collected and stored at −80°C for later use. Animal care and procedures were performed in accordance with the Laboratory Animal Care Guidelines approved by the Medical Ethics Committee of Xi'an Jiaotong University.

### 2.8. Statistical Analysis

The statistical analyses were performed using the SPSS 13.0 software. All values were presented as mean ± S.E.M. The statistical significance between two groups was tested by one-way analysis of variance (ANOVA) followed by Tukey's test. The statistical significance was set at *P* ≤ 0.05.

## 3. Results and Discussion

In the recent years, curcumin has been extensively investigated for its therapeutic value. Its anti-inflammatory effect which is equivalent to that of steroidal and nonsteroidal drugs, e.g., indomethacin and phenylbutazone, is one of the most studied properties [8, 12]. In various inflammation-related chronic illnesses such as cardiovascular disease, cancer, diabetes, and obesity, curcumin has shown good therapeutic effects [13]. The pivotal role of inflammation in the pathogenesis of atherosclerosis has been documented. We previously reported that aldosterone exerted its proinflammation effect on VSMCs by inducing CRP generation [7]. Here, we explored whether curcumin could inhibit this effect.

**Figure 1:**
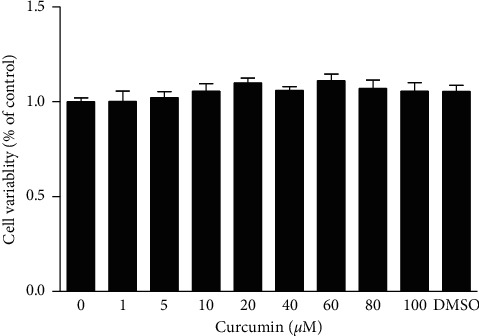
Effect of curcumin on the viability of VSMCs.

The effect of curcumin at different concentrations on the viability of VSMCs was determined using MTT assay. The results revealed that incubation of the cells with curcumin at 1–100 *μ*mol/L for 24 h did not affect the viability of VSMCs (Figure 1). Therefore, the cytotoxicity of curcumin was considered negligible.

VSMCs were incubated for 24 h with 1–100 *μ*mol/L curcumin. Then, the cellular viability was assayed by the MTT method. DMSO (0.1%) was used as solvent control. Data were expressed as mean ± S.E.M (*n* = 3).

**Figure 2:**
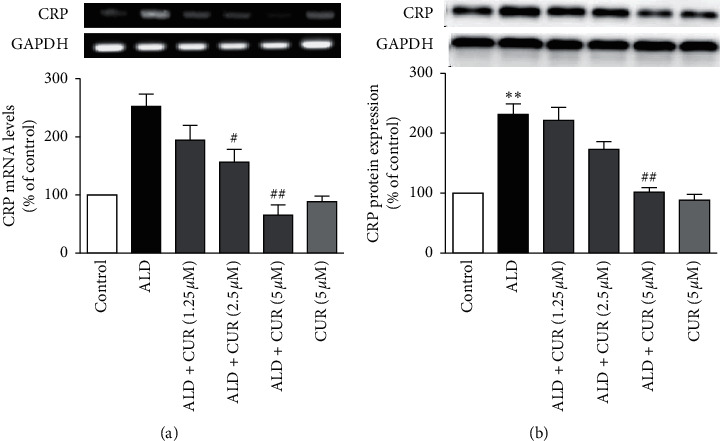
Effect of curcumin on aldosterone-stimulated CRP expression in VSMCs.

RT-PCR and western blotting results showed that treatment of cells with 10 nmol/L aldosterone for 24 h increased mRNA and protein expression of CRP in VSMCs significantly (*P* < 0.01 vs. control). Pretreatment of the cells with curcumin at 1.25, 2.5, and 5 *μ*mol/L prior to stimulation with aldosterone canceled the elevation of CRP mRNA and protein caused by aldosterone in a concentration-dependent way (*P* < 0.05 or *P* < 0.01 vs. aldosterone alone). Curcumin alone did not affect the basal expression of CRP in VSMCs (Figures 2(a) and 2(b)). These results indicated that curcumin decreases CRP generation in VSMCs *in vitro* to alleviate inflammation.

VSMCs were preincubated with curcumin (CUR) for 1 h before the stimulation with 10 nmol/L aldosterone (ALD) for 24 h. (a) CRP mRNA expression was detected by RT-PCR and (b) CRP protein expression was identified by western blotting. CUR alone (5 *μ*mol/L) acted as a drug control. Data were expressed as means ± S.E.M (*n* = 3). ^*∗∗*^*P* < 0.01 vs. control; #*P* < 0.05 and ##*P* < 0.01 vs. ALD alone.

**Figure 3:**
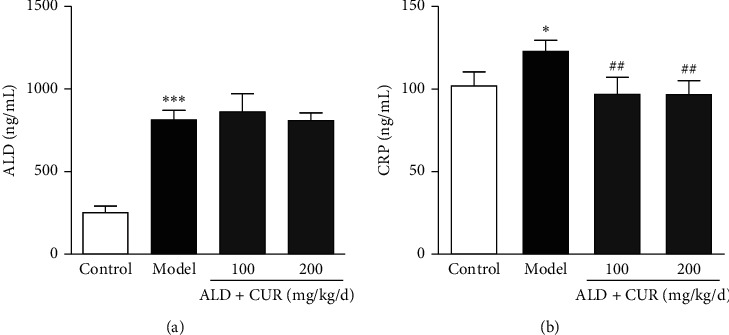
Effects of curcumin on serum aldosterone and CRP levels of hyperaldosteronism rats.

To confirm the inhibitory effect of curcumin *in vivo*, a rat model of hyperaldosteronism was established. Serum aldosterone and CRP levels in rats with hyperaldosteronism were significantly elevated (*P* < 0.05 or *P* < 0.001 vs. control), while body weight and SBP were unaffected (data not shown). Compared with rats with hyperaldosteronism, rats receiving curcumin intervention exhibited lower serum CRP level (*P* < 0.01 vs. model, Figures 3(b)), but serum aldosterone concentration was similar between the hyperaldosteronism groups (Figure 3(a)). These results revealed that curcumin reduced production of CRP but not aldosterone secretion or other metabolic variables.

Aldosterone (ALD, 0.75 *μ*g/h) was infused into rats for 4 weeks with a subcutaneous osmotic minipump. Simultaneously, rats received curcumin (CUR) via an intragastrical way. At the end of the experiment, blood was collected to detect the serum concentrations of ALD (a) by radioimmunoassay and CRP (b) by ELISA. Results were expressed as mean ± S.E.M (*n* = 5). ^*∗*^*P* < 0.05 and ^*∗∗∗*^*P* < 0.001 vs. control; ##*P* < 0.01 vs. model group.

**Figure 4:**
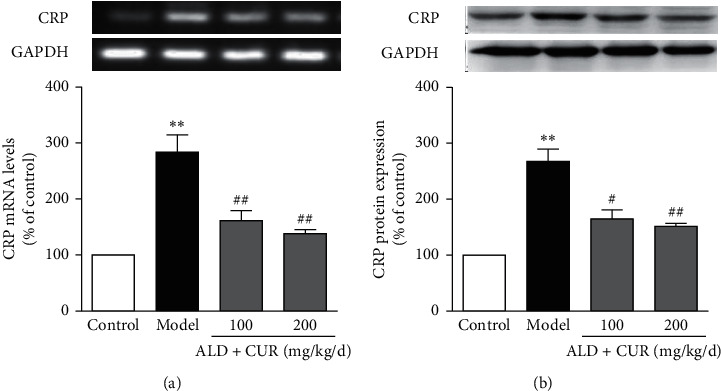
Effect of curcumin on CRP expression in the thoracic aorta of hyperaldosteronism rats.

Further tests showed that mRNA and protein levels of CRP in the thoracic aorta of hyperaldosteronism rats were significantly higher than in control rats (*P* < 0.01 vs. control). Simultaneous administration of curcumin downregulated CRP expression in the thoracic aortic wall at mRNA and protein levels (*P* < 0.05 or *P* < 0.01 vs. model, Figures 4(a) and 4(b)). These results indicated that curcumin diminished aldosterone-induced CRP mRNA and protein expression in the thoracic aorta *in vivo*.

Protocol for administration of aldosterone (ALD) and curcumin (CUR) is the same as in Figure 3. The thoracic aorta was removed at the end of the experiment. CRP mRNA (a) and protein (b) expression were examined by RT-PCR and western blotting, respectively. Results were expressed as mean ± S.E.M (*n* = 5). ^*∗∗*^*P* < 0.01 vs. control; #*P* < 0.05 and ##*P* < 0.01 vs. model group.

**Figure 5:**
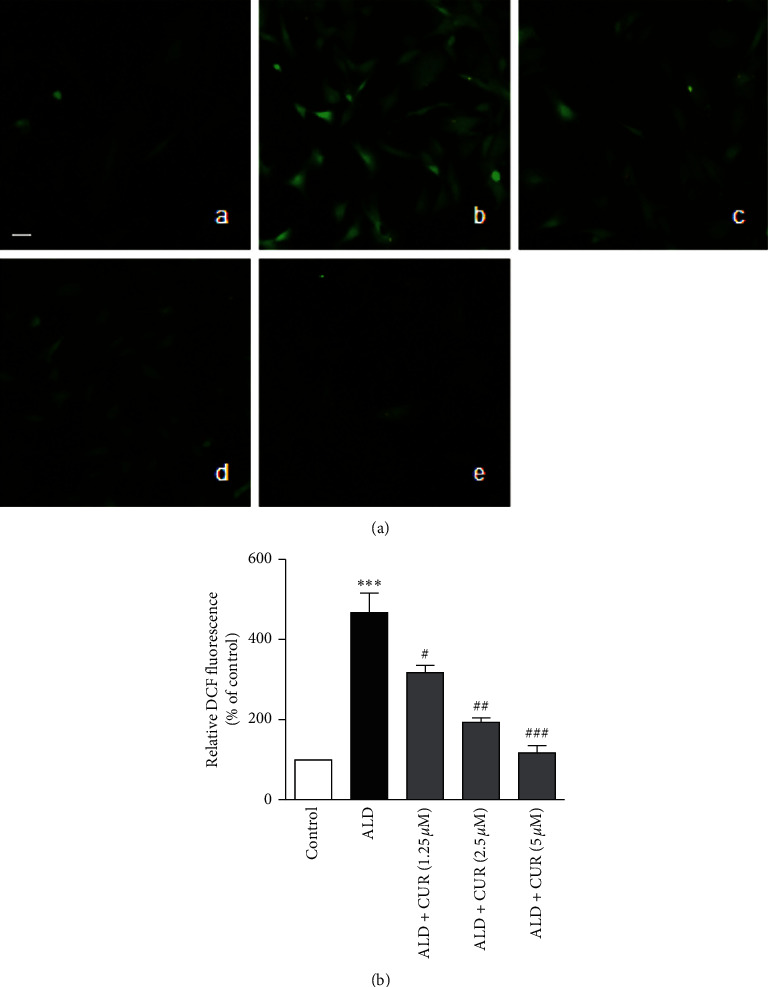
Effect of curcumin on aldosterone-stimulated ROS production in VSMCs.

We previously reported that eplerenone inhibited ALD-induced CRP generation by interfering with the ROS-ERK1/2 signaling pathway [14]. In the present study, the effects of curcumin on ROS production and ERK1/2 phosphorylation were examined. Oxidative stress takes a crucial part in the pathogenesis of atherosclerosis. Besides its direct oxidative damage, ROS also causes cell injury by evoking inflammatory reactions [15]. Our previous study proved that intracellular ROS is involved in the aldosterone-induced CRP generation in VSMCs [7]. To probe the inhibitory mechanism of curcumin on aldosterone-induced CRP expression, ROS production in the cells was determined. VSMCs treated with aldosterone at 10 nmol/L showed ROS levels higher than those in the control group (*P* < 0.001). Pretreatment of the cells with curcumin at 1.25, 2.5, and 5 *μ*mol/L reduced aldosterone-stimulated ROS production in a concentration-dependent way (*P* < 0.05, *P* < 0.01 or *P* < 0.001 vs. aldosterone alone, Figures 5(a) and 5(b)). Collectively, these results showed that curcumin exerted antioxidative effect by diminishing aldosterone-stimulated ROS production in VSMCs which is consistent with previous reports [16], suggesting that curcumin reduced aldosterone-stimulated CRP generation in VSMCs by scavenging ROS.

VSMCs were pretreated with curcumin (CUR) for 1 h followed by stimulation with 10 nmol/L aldosterone (ALD) for 2 h. Then, the cells were incubated with H_2_DCF-DA (10 *μ*mol/L) for 1 h. Finally, the cells were observed, and the pictures were captured under a fluorescence microscope. The fluorescent intensity was measured by using a fluorescence microscope. (a) Representative fluorescence images: (A) control, (B) ALD, (C) ALD + 1.25 *μ*mol/L CUR, (D) ALD + 2.5 *μ*mol/L CUR, and (E) ALD + 5 *μ*mol/L CUR. (b) Relative fluorescence intensity quantified from the fluorescence images. Data were expressed as mean ± S.E.M (*n* = 3). ^*∗∗∗*^*P* < 0.001 vs. control; #*P* < 0.05, ##*P* < 0.01 and ###*P* < 0.001 vs. ALD alone. Scale bar, 100 *µ*m. Original magnification, ×200.

**Figure 6:**
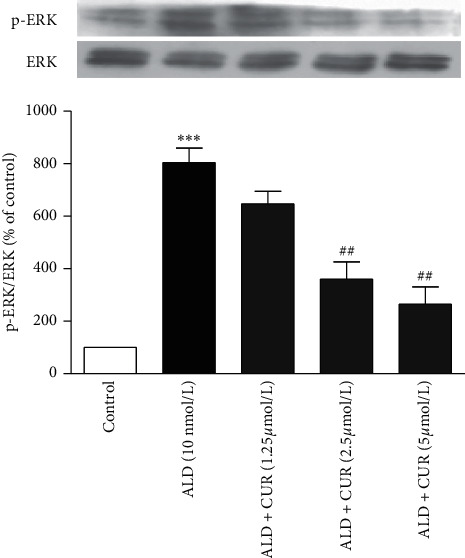
Effect of curcumin on aldosterone-activated ERK1/2 phosphorylation in VSMCs.

To exert anti-inflammation activity, curcumin targets the activity of protein kinases [8]. Given the impact of ERK1/2 phosphorylation in aldosterone-induced CRP expression [7], the effect of curcumin on aldosterone-activated ERK1/2 phosphorylation was examined. The result revealed that aldosterone activated ERK1/2 phosphorylation in VSMCs (*P* < 0.001 vs. control), while pretreatment of the cells with curcumin at the concentrations shown above diminished this effect (*P* < 0.01 vs. aldosterone alone, Figure 6).

VSMCs were stimulated with 10 nmol/L aldosterone (ALD) for 30 min after pretreatment with curcumin (CUR) for 1 h. Then, the ERK1/2 phosphorylation level was detected by western blotting. Data were expressed as mean ± S.E.M (*n* = 3). ^*∗∗∗*^*P* < 0.001 vs. control; ##*P* < 0.01 vs. ALD alone.

**Figure 7:**
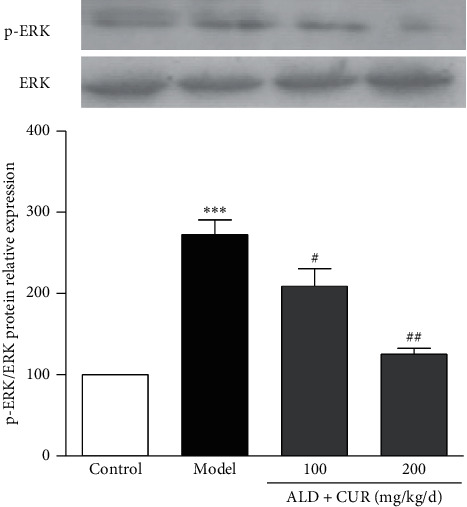
Effect of curcumin on ERK1/2 phosphorylation in the thoracic aorta of hyperaldosteronism rats.

The phosphorylation of ERK1/2 in the thoracic aorta of hyperaldosteronism rats was upregulated as well (*P* < 0.001 vs. control). In comparison with the model group, ERK1/2 phosphorylation was downregulated in the thoracic aorta of curcumin-treated groups (*P* < 0.05 or *P* < 0.01, Figure 7). According to these results, we conclude that curcumin diminished aldosterone-activated ERK1/2 phosphorylation in VSMCs both *in vitro* and *in vivo*. This finding is consistent with previous reports that curcumin reduces the production of inflammatory cytokines by interfering with the MAPK signaling pathway [17]. ROS mediates aldosterone-activated ERK1/2 phosphorylation leading to the production of CRP in VSMCs [7]. Together with previous reports, curcumin inhibits aldosterone-induced CRP generation in VSMCs by interfering with the ROS-ERK1/2 signaling pathway. Whether there are other mechanisms through which curcumin exerts anti-inflammatory effects in ALD-induced inflammation in VSMCs is a possibility requiring further studies.

The protocol for administration of aldosterone (ALD) and curcumin (CUR) is the same as in Figure 3. At the end of the experiment, the thoracic aorta was removed, and the total protein was extracted to determine ERK1/2 phosphorylation by western blotting. Results were expressed as mean ± S.E.M (*n* = 5). ^*∗∗∗*^*P* < 0.001 vs. control; #*P* < 0.05 and ##*P* < 0.01 vs. model group.

The concentration we used in the *in vitro* experiment is much higher than the plasma concentration in the *in vivo* experiment if bioavailability and metabolism are taken into account [18]. The long-term effect of curcumin at low doses *in vitro* and the effects of curcumin agents with higher bioavailability *in vivo* remain to be investigated.

## 4. Conclusions

The present study shows that curcumin suppresses aldosterone-induced CRP generation in VSMCs by interfering with the ROS-ERK1/2 signaling pathway. These results reveal a mechanism through which curcumin represses inflammation and confers cardiovascular protection. Our findings further confirm the anti-inflammatory and cardiovascular protective effects of curcumin and suggest its potential clinical use in cardiovascular inflammation.

## Data Availability

All the data used to support the findings of this study are included within the article.

## References

[1] Libby P., Okamoto Y., Rocha V. Z., Folco E. (2010). Inflammation in atherosclerosis:. *Circulation Journal*.

[2] Ansar W., Ghosh S. (2013). C-reactive protein and the biology of disease. *Immunologic Research*.

[3] Torzewski M., Rist C., Mortensen R. F. (2000). C-reactive protein in the arterial intima. *Arteriosclerosis, Thrombosis, and Vascular Biology*.

[4] Ames M. K., Atkins C. E., Pitt B. (2019). The renin-angiotensin-aldosterone system and its suppression. *Journal of Veterinary Internal Medicine*.

[5] Namsolleck P., Unger T. (2014). Aldosterone synthase inhibitors in cardiovascular and renal diseases. *Nephrology Dialysis Transplantation*.

[6] Farquharson C. A. J., Struthers A. D. (2002). Aldosterone induces acute endothelial dysfunction in vivo in humans: evidence for an aldosterone-induced vasculopathy. *Clinical Science*.

[7] Zhang X. (2014). Aldosterone induces C-reactive protein expression via MR-ROS-MAPK-NF-kappaB signal pathway in rat vascular smooth muscle cells. *Molecular and Cellular Endocrinology*.

[8] Fernández-Moriano C., González-Burgos E., Gómez-Serranillos M. P. (2019). Curcumin: current evidence of its therapeutic potential as a lead candidate for anti-inflammatory drugs—an overview. *Natural Product Drug Discovery 2019*.

[9] Pang X., Liu J., Zhao J. (2014). Homocysteine induces the expression of C-reactive protein via NMDAr-ROS-MAPK-NF-*κ*B signal pathway in rat vascular smooth muscle cells. *Atherosclerosis*.

[10] Wang C.-H., Li S.-H., Weisel R. D. (2003). C-reactive protein upregulates angiotensin type 1 receptors in vascular smooth muscle. *Circulation*.

[11] Hilgers K. F., Hartner A., Porst M., Veelken R., Mann J. F. E. (2001). Angiotensin II type 1 receptor blockade prevents lethal malignant hypertension. *Circulation*.

[12] Menon V. P., Sudheer A. R. (2007). Antioxidant and anti-inflammatory properties of curcumin. *Advances in Experimental Medicine and Biology*.

[13] Shimizu K., Funamoto M., Sunagawa Y. (2019). Anti-inflammatory action of curcumin and its use in the treatment of lifestyle-related diseases. *European Cardiology*.

[14] Zhang X., Liu J., Pang X., Zhao J., Xu S., Zhao J. (2017). Eplerenone inhibits aldosterone-induced CRP generation in rat vascular smooth muscle cells by regulating the MR-ROS-ERK1/2 signal pathway. *European Journal of Inflammation*.

[15] Park H. S., Kim S. R., Lee Y. C. (2009). Impact of oxidative stress on lung diseases. *Respirology*.

[16] Meng Z., Yan C., Deng Q., Gao D.-F., Niu X.-L. (2013). Curcumin inhibits LPS-induced inflammation in rat vascular smooth muscle cells in vitro via ROS-relative TLR4-MAPK/NF-*κ*B pathways. *Acta Pharmacologica Sinica*.

[17] Shi X., Zheng Z., Li J. (2015). Curcumin inhibits A *β*-induced microglial inflammatory responses in vitro: involvement of ERK1/2 and p38 signaling pathways. *Neuroscience Letters*.

[18] Pan M. H., Huang T. M., Lin J. K. (1999). Biotransformation of curcumin through reduction and glucuronidation in mice. *Drug Metabolism and Disposition: The Biological Fate of Chemicals*.

